# Yeast rises to the occasion

**DOI:** 10.7554/eLife.00933

**Published:** 2013-06-18

**Authors:** Mark A Ragan

**Affiliations:** 1 **Mark A Ragan** is at the Institute for Molecular Bioscience and the School of Information Technology and Electrical Engineering, University of Queensland, Brisbane, Australiam.ragan@uq.edu.au

**Keywords:** regulatory evolution, duplication, divergence, carbon lifestyle, module, gene expression, S. cerevisiae, S. pombe

## Abstract

Genetic analyses of 15 species of yeast have shed new light on the divergence of gene regulation during evolution, with significant changes occurring after an event in which a whole genome was duplicated.

**Related research article** Thompson D, Roy S, Chan M, Styczynsky M, Pfiffner J, French C, Socha A, Thielke A, Napolitano S, Muller P, Kellis M, Konieczka JH, Wapinski I, Regev A. 2013. Evolutionary principles of modular gene regulation in yeasts. *eLife* **2**:e00603. doi: 10.7554/eLife.00603

**Image** The 15 species of yeast studied by Thompson et al.

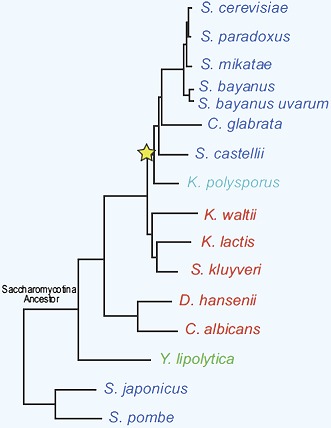

One of the less-anticipated outcomes from the past decade of genomics is how poorly the complexity of organisms correlates with their gene number. Even allowing for a large measure of anthropocentrism, it remains puzzling that humans have only about 20,800 protein-coding genes, whereas water fleas have about 30,900 and the rice plant has 40,000 or so. Working with this pedestrian number of genes, the human body nonetheless gives rise to more than 400 specialised cell types (Vickaryous and Hall, 2006), and the remarkable diversity of these cell types in terms of form and function arises from different sets of genes (known as modules) being co-expressed at different times.

We understand quite a lot about how genes themselves evolve and diversify, but we know very much less about the evolution of the processes that regulate the expression of genes. Are modules stable over evolutionary timescales, or are they assembled opportunistically as required? Are duplicate copies of genes retained in the ancestral module or are they reassigned to another module? And if they are reassigned, do they tend to be reassigned to the same module or to different modules, and does this happen shortly after duplication or does it continue over a much longer time? Does it matter whether the duplicates were generated sporadically or via whole-genome duplication? Is regulatory evolution driven by natural selection, and does it correlate with changes in lifestyle or the copy number of chromosomes? Indeed, are there general principles of modular gene regulation, or is the story of gene regulation one of contingency and anecdote?

Writing in *eLife*, Dawn Thompson, Aviv Regev and co-workers—including Thompson and Sushmita Roy as joint first authors—report the results of a large-scale assault on these questions. Thompson et al. focused on 15 species of yeast for which complete genome sequences are known (Figure 1). Yeasts offer many advantages for studying the evolution of gene regulation, over and above their well known genetics. In particular, their evolutionary history over the past 300 million years is well known and is comparable to those of teleost fishes (Near et al., 2012) and seed plants (Clarke et al., 2012) in terms of its depth. Their physiology is also well understood, but it is also surprisingly diverse: for example, different species of yeast colonise different ecological niches, utilise a range of different carbon sources, and differ in their preference for oxidative phosphorylation vs a more fermentative lifestyle.

**Figure 1.**
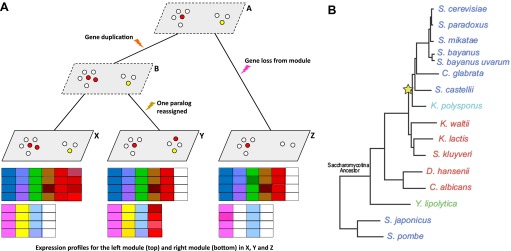
Given gene expression profiles for a number of species, and a gene tree and a species tree for these species, an algorithm called Arboretum (Roy et al., 2013) can be used to determine how sets of genes called modules have evolved over the period covered by the these trees. (**A**) Schematic diagram showing gene expression profiles (bottom) for two gene modules in three different species (**X**, **Y** and **Z**); the left module contains 5 or 6 genes in these species, while the right module contains 2, 3 or 4 genes. **A** and **B** are the inferred ancestral states of these modules. A local gene duplication event along the lineage **A** → **B** results in duplicates (paralogs) of the red gene in the left module: one or both of these duplicates can be retained or assigned to a different module; both are retained in the module along **B** → **X**, and one is reassigned along **B → Y**. Genes can also be lost (**A** → **Z**) or gained by modules. (**B**) Thompson et al. studied 15 species of yeast; 13 of these are descended from a single Saccharomycotina species, and a whole genome duplication event (yellow star) resulted in seven of the species. See Figure 1A of Thompson et al. for full details.

Moreover, as a bonus, the 15-yeast dataset compiled by Thompson et al. spans a whole-genome duplication event that has affected seven of the 15 species (Figure 1; Wolfe and Shields, 1997; Kellis et al., 2004). By simultaneously duplicating all genes and their regulatory elements, even the most ancestral elements, whole-genome duplication events make it possible for a lineage to explore modes of gene regulation that would not become accessible as a result of sporadic, localised duplication events (Lynch and Katju, 2004).

Patterns of gene co-expression are sometimes conserved over substantial timescales, despite significant turnover in the associated transcription factors and chromatin organisation. Sometimes this turnover is coupled to adaptive changes in lifestyle, whereas other changes in regulation may be neutral, analogous to the genetic drift that happens at the sequence level (Tsankov et al., 2011; Baker et al., 2012). However, the intrinsic technical difficulty of these experiments, coupled with physiological diversity displayed by different species of yeast, has until now made it difficult to test the generality of these findings.

Focusing on growth in glucose and its depletion in batch culture, Thompson, Regev and co-workers—who are based at the Broad Institute of MIT and Harvard, and also at MIT—began by devising a medium that supports the growth of all 15 yeasts at comparable rates (Thompson et al., 2013). They next identified six physiologically comparable time-points along the growth curve of each yeast. Gene expression profiles confirmed that these six time-points were indeed physiologically comparable. Thompson et al. then used oligomer arrays to profile the transcriptome of each yeast species at each time point.

To compare expression profiles and track the assignment of each gene to one or another module along the phylogenetic tree, the Broad-MIT team developed a probabilistic algorithm (called Arboretum) that delineates the modules (which can change size and composition over time) and computes the trajectory of every gene through a module in each extant and inferred ancestral species (see Figure 1A; Roy et al., 2013). Thompson et al. used Arboretum to map the evolution of functional annotation, *cis*-regulatory motifs and nucleosome-free regions within and across modules for single-copy genes, and also for duplicates arising from sporadic or whole-genome duplication.

Many key questions yield to this systematic approach. Two-thirds of the variation in transcriptional response is captured by five expression modules. Genes are conserved within these modules in a way that is inversely proportional to evolutionary time, with two modules (those related to growth and stress-response functions) being more conservative than the other three. Gene reassignment between modules is often consistent with changes in lifestyle or the copy number of chromosomes. Duplicates are more likely to be reassigned than single-copy genes, with this reassignment often occurring in a brief ‘window of opportunity’ after duplication; however, duplicates that arise from the whole-genome duplication continue to be reassigned over a much longer time. Neo-functionalization (where one copy is retained, the other reassigned) and symmetric divergence (both copies reassigned to the same module) are more frequent than asymmetric divergence (reassignment to different modules).

Do any of these observations look like a principle? They all seem to apply regardless of gene function, lifestyle or evolutionary distance on the yeast tree. In an experiment with eight of these species, many of the same responses were seen in response to heat shock. Finally, Thompson, Regev and co-workers point to fascinating similarities between the up-regulation of genes for nucleotide salvage and glycine synthesis at one of the six time-points they study (when the available glucose has been depleted) in some yeasts, and regulatory changes involving the same pathways in cancer cells that show an effect called the Warburg effect.

Yeast is one of the less-complex eukaryotes, but the emergence of evolutionary principles for gene regulation in these experiments represents another addition to the list of unanticipated outcomes of genomic biology.
